# Feasibility, Tolerance, and Quality of Life for Hypofractionation Versus Conventional Fractionation for Post-mastectomy Radiotherapy in Indian Patients

**DOI:** 10.7759/cureus.23497

**Published:** 2022-03-25

**Authors:** Deepika Malik, Ashok Singh, Manoj M Birajdar, Virendra J Vyas

**Affiliations:** 1 Radiation Oncology, Maharishi Markandeshwar Institute of Medical Sciences and Research (MMIMSR), Ambala, IND; 2 Radiation Oncology, Mahatma Gandhi Institute of Medical Sciences, Wardha, IND; 3 Radiation Oncology, All India Institute of Medical Sciences, Raipur, IND; 4 Clinical Oncology, Indian Institute of Head and Neck Oncology, Indore, IND

**Keywords:** post-mastectomy radiotherapy, breast cancer, quality of life, acute toxicity, hypofractionation

## Abstract

Introduction: The international standard for post-operative radiotherapy for breast cancer delivers hypofractionated radiotherapy. However, many centers in India still follow the longer conventional schedule probably because of paucity of large prospective trials in Indian patients on the same and apprehension regarding tolerance of high dose per fraction in the said population. We aimed to test the feasibility of hypofractionation in our setting and compared the toxicities and the quality of life in patients receiving conventional and hypofractionated radiotherapy.

Materials and methods: Eighty histopathologically proven women of non-metastatic carcinoma breast who underwent modified radical mastectomy were assigned to receive 50 Gray/25 fractions/five weeks or 40 Gray/15 fractions/three weeks. Patients were assessed for the following toxicities - radiation dermatitis, radiation pneumonitis, dysphagia, skin fibrosis, lymphedema, shoulder stiffness, and brachial plexopathy, during radiation and at treatment completion and then at first, third, and sixth-month follow-up. Health-related quality of life was assessed using the European Organisation for Research and Treatment of Cancer core quality of life questionnaire (EORTC QLQ-C30) and breast cancer-specific quality of life questionnaire (QLQ-BR23) at treatment completion and then at first, third, and sixth-month follow-up.

Results and conclusion: We had a mean follow-up of 12.78 months. All the assessed toxicities and quality of life scores were comparable between the two arms at all time points of evaluation (p>0.05); 40 Gray in 15 fractions over three weeks is feasible and as safe as the five-week schedule with comparable quality of life. Hypofractionation can be practiced as a routine for post-mastectomy breast cancer patients as this shorter radiotherapy schedule is convenient and cheaper for the patients with no compromise on normal tissue toxicity or quality of life.

## Introduction

The international standard radiotherapy regimen after breast-conserving surgery or mastectomy for breast cancer delivers hypofractionated radiotherapy after it was proven to be as efficacious to the conventional fractionation of 50 Gray (Gy) in 25 fractions over five weeks with similar adverse reactions. Post-operative irradiation reduces locoregional recurrence risk by about 70% and reduces breast cancer mortality and also improves overall survival [1,2].

Hypofractionation, a strategy allowing shortening of overall treatment time, involves higher fraction doses (above 2 Gy), such that a biologically equivalent dose can be delivered in fewer fractions. Radiobiology justifies hypofractionation in breast cancer owing to a low α/β value at 4 Gy which is comparable to that of healthy tissues (α/β is 3.5 Gy for soft tissues) [3]. Thus, a higher dose per fraction is assumed to be more efficient in destroying tumor cells with concerns however of a high incidence and severity of late radiation toxicity.

The landmark UK Standardisation of Breast Radiotherapy (START) trials for hypofractionation (START-A, 50 Gy in 25 fractions over five weeks compared with 41.6 Gy or 39 Gy in 13 fractions over five weeks and START-B, 50 Gy in 25 fractions over five weeks compared with 40 Gy in 15 fractions over three weeks) in their five and 10-year follow-up results showed that hypofractionated radiotherapy schedule oﬀered rates of local-regional tumor relapse and late adverse eﬀects at least as favorable as the standard schedule of 50 Gy in 25 fractions [4-6]. Hypofractionation is being practiced routinely for breast cancer in the UK and many parts of the world. However, in India, some centers still practice the five-week schedule routinely despite the robust evidence the UK START trials provided in favor of hypofractionation. This could probably be because of apprehension regarding the inferiority of hypofractionation with respect to toxicities as sadly there are no large prospective randomized trials conducted in India on this aspect. Also, a major part of the patient population studied in the UK trials as well as other studies on hypofractionation in breast cancer had post breast conservation surgery patients. Post-mastectomy patients formed a small part of these studies. Therefore, to some extent, the above-mentioned apprehension is justified until hypofractionation is studied in the target population where it is intended to be used.

India is currently witnessing what could be called an epidemic of cancer, breast cancer being a major attributor in females. Breast cancer has now become the most common cancer, with 2,261,419 new cases diagnosed worldwide and 178,361 new cases diagnosed in India in 2020. Breast cancer accounts for 13.5% of all cancer cases in India, compared to 11.77% worldwide [7]. This burden is likely to grow not only in terms of the absolute number of cases but also in terms of incidence owing to the urbanization and changing lifestyle patterns of the Indian population [8]. The proportion of breast cancers registered annually at our center reinforces this estimate (11% to 24% over a span of 20 years from the year 1998 to 2018).

This rising breast cancer incidence has a direct bearing on the burden on radiotherapy machines [8]. As per the World Health Organisation, a developing country should have at least one teletherapy machine for a population of one million. Going by these figures, India has to increase the number of existing teletherapy machines by fourfold [9]. In 2014, policy analysis on cancer research in India said that research on more cost-effective treatment pathways that will provide good outcomes is needed in India. The authors mentioned that results of clinical research on brachytherapy, hypofractionated radiotherapy schedules, and regular repeating (metronomic) chemotherapy regimens to reduce treatment times can drive down the costs of care and enable more patients to be effectively treated [10].

Conventional fractionation of five weeks requires a long in or near hospital stay or commuting to a hospital for this long period. By adopting hypofractionation, the ever-rising burden on radiotherapy machines and the consequential long waiting lists can be controlled. This would be of great logistic benefit to resource-limited radiotherapy centers which are the backbone of cancer care in India. A shorter course of radiotherapy would also relatively relieve poor patients financially by reducing the number of days of treatment, travel to the radiotherapy center, or staying in the hospital premises. Also, cost of the radiotherapy treatment of three weeks would cost 0.6 times lesser than a five-week schedule. The purpose of this study was therefore to determine the feasibility and tolerability of hypofractionation and its effect on health-related quality of life in our patients over the tries and tested conventional radiotherapy schedule.

## Materials and methods

Patients

After obtaining approval from the institutional ethics committee (ref. no.: MGIMS/IEC/RADTH/98/2014) and written informed consent from patients, 80 histopathologically proven patients of non-metastatic breast cancer, treated surgically with modified radical mastectomy (MRM), and having indication for post-mastectomy radiotherapy were enrolled from the department of radiation oncology at a rural medical college in Maharashtra between January 2015 and December 2017. Patients above 65 years of age, Karnofsky Performance Status (KPS) of 70 or less, with a history of prior radiotherapy to chest or neck were excluded from the study. All patients underwent a thorough history and physical examination and relevant blood-workup, chest radiograph, and abdominal ultrasonography. All patients were evaluated for hormone receptor status - estrogen receptor (ER), progesterone receptor (PR), and human epidermal growth factor receptor 2 (HER2/neu) status. Computed tomography chest and abdomen and bone-scan were done for stage III or beyond or on clinical or biochemical suspicion of metastasis. The 80 patients enrolled were randomized by block method into two arms. Forty patients in the study arm received 40.05 Gray in 15 fractions with a fraction size of 2.67 Gray, once daily for five days a week over three weeks (hypofractionated arm). Forty patients in the control arm received 50 Gray in 25 fractions with a fraction size of two Gray, once daily for five days a week over five weeks (conventional arm).

Radiation technique

Patients lay supine on an adjustable carbon fiber breast board with ipsilateral arm abducted and externally rotated and fixed on strategically placed adjustable handgrip and neck hyperextended to opposite side. All patients were simulated on Nucletron Simulix Evolution (Stockholm, Sweden: Elekta). Chest-wall was treated with medial and lateral tangential portals with superior border placed at the level of head of clavicle, inferior border 2 cm below the contralateral inframammary fold, medial border at patient midline, and lateral border at mid-axillary line attempting to cover the MRM scar wherever possible, with no bolus. Supraclavicular fossa (when indicated) was treated with a single anterior portal with superior border at thyroid-cricoid notch, inferior border at the superior border of chest wall portals with a 5 mm gap on skin surface between the two portals, medial border 1 cm lateral to midline, and lateral border at the junction of medial two-thirds and lateral one-third of the clavicle. A conventional two-dimensional plan was created and patients were treated on cobalt-60 (Theratron Phoenix; Ontario, Canada: Best Theratronics Ltd.) machine. Dose to chest-wall was prescribed at midpoint of chest-wall contour and at D-max for supraclavicular portal.

Assessment of outcomes

Toxicities compared between the two arms were skin toxicity, lung toxicity, dysphagia, shoulder restriction (graded as per Radiation Therapy Oncology Group {RTOG} morbidity schema), brachial plexopathy (clinical finding said to be present if patient had pain, numbness, paresthesia, and motor weakness), and lymphedema (clinical finding using tape measurement said to be present if a difference of more than 2 cm between arm circumference measured at baseline and at evaluation between affected and contralateral arm; arm circumference measured at 10 cm above and 10 cm below olecranon process) [11]. Patients were assessed for the toxicities at radiotherapy completion, and then at one month, three months, and six months after completion of radiotherapy, ensuring that as far as possible toxicity was assessed and documented on a standard scoring chart by a single observer (the principal investigator) and photographic evidence for skin reactions were obtained for comparison at follow-up. Skincare and physiotherapy were emphasized from day one of treatment and then at subsequent visits.

Quality of life was assessed using the European Organisation for Research and Treatment of Cancer core quality of life questionnaire (EORTC QLQ-C30) and breast cancer-specific quality of life questionnaire (QLQ-BR23) which were filled by all patients at baseline, at treatment completion, and then at one month, three months, and six months follow-up [12]. Questionnaires were provided in patients' own language (Marathi/Hindi/English) and for illiterate ones, questions from the questionnaires were read out by the same departmental social worker for all such patients (to avoid bias). It was ensured that all patients answered all questions in the questionnaire. Scoring was done in accordance with the EORTC scoring manual.

Conclusions on comparison of efficacy of radiotherapy schedules could not be drawn from this study owing to a short follow-up as only a limited time was available for conducting this study. Therefore response assessment in form of local recurrence or distant metastases has not been taken as an objective for this study. Patient compliance was assessed by analyzing the unplanned gaps in radiotherapy regimen. Gaps in radiotherapy were labeled as unplanned when they were caused by factors other than public holidays, statutory holidays, or machine breakdown.

Statistical analysis

Statistical analysis was done using IBM SPSS Statistics version 12.0 (Chicago, IL: SPSS Inc.). The categorical variables were presented as mean (standard deviation), and Pearson’s chi-square test or Fisher’s exact test was used as the test of significance. The continuous variables were presented as frequency (percentage) and unpaired t-test was used as the test of significance. The equality of variance for comparison of means was tested using Levene’s test. To calculate probability of radiation-induced dermatitis and pneumonitis we used life-table analysis. P-value<0.05 was considered statistically significant.

## Results

The mean age of our study population was 45.88 years (range: 28-65 years). Most patients (i.e., 41.25%, 33 out of 80 patients) belonged to stage IIIA. A total of 52.5% (i.e., 42 out of 80) were left-sided breast cancers. Also, 65% of patients (i.e., 52 out of 80 patients) were hormone receptor-positive, out of which 86.53% (i.e., 45 out of 52) were estrogen receptor (ER) positive and the rest were progesterone receptor (PR) positive. A total of 35% of patients (i.e., 28 out of 80 patients) were HER2/neu positive. And 18.75% of patients (i.e., 15 out of 80 patients) were triple-negative breast cancers. Patient population characteristics were well balanced between the two arms (Table 1).

**Table 1:** Population characteristics of the two arms at baseline. KPS: Karnofsky Performance Status; HER2/neu: human epidermal growth factor receptor 2; ER: estrogen receptor; PR: progesterone receptor

Parameter			Hypofractionation arm	Conventional arm	p-Value
Mean age, years			44.88	46.88	0.32
KPS, mean (SD)			85.75 (5.9)	86.0 (5.9)	0.85
Stage of presentation, frequency (%)	II A		3 (7.5)	3 (7.5)	1.00
	II B		13 (32.5)	13 (32.5)	
	III A		16 (40)	17 (42.5)	
	III B		2 (5)	1 (2.5)	
	III C		6 (15)	6 (15)	
Laterality, frequency (%)	Left		23 (57.5%)	19 (47.5%)	0.37
	Right		17 (42.5%)	21 (52.5%)	
ER positive, frequency (%)			20 (50%)	25 (62.5%)	0.37
PR positive, frequency (%)			15 (37.5%)	17 (42.5%)	0.82
HER2/neu positive, frequency (%)			15 (37.5%)	13 (32.5%)	0.82
Menstrual status, frequency (%)		Pre-menopausal	18 (45%)	11 (27.5%)	0.16
		Post-menopausal	22 (55%)	29 (72.5%)	
Duration of follow-up (months) mean (SD)			12.1 (4.3)	12.9 (4.1)	0.38

Forty patients in each arm completed the treatment. The mean follow-up duration for this study was 12.48 months. One patient in hypofractionated arm was lost to follow-up after one month of follow-up, and one patient in conventional arm died due to acute myocardial infarction two months after completing radiotherapy.

Toxicity comparison

At the end of radiotherapy, 62.5% and 20% of patients in hypofractionation arm had grade I and II skin toxicity, respectively compared to 57.5% and 32.5% of patients in conventional arm (p=0.35) while none had grade III or beyond in both arms. At one month after completion, 82.5% of patients had grade I skin toxicity in hypofractionation arm, compared to 90% with grade I and 5% with grade II in conventional arm (p=0.09), and none had grade II or beyond in hypofractionation arm and grade III or beyond in conventional arm. At three months 43.6% and 48.7% of patients had grade I skin toxicity in hypofractionation and conventional arm respectively (p=0.82), with none having grade II or beyond. At six months 20.5% and 28.2% of patients had grade I skin toxicity (p=0.60), none having grade II or beyond (Table 2).

**Table 2:** Comparison of radiation dermatitis. The table shows comparison of radiation dermatitis at different time points of evaluation between conventional radiotherapy arm and hypofractionated radiotherapy arm. The grading of radiation dermatitis is as per the RTOG acute toxicity grading. RT dermatitis: radiation dermatitis; HF: hypofractionated arm; CV: conventional fractionation arm; RTOG: Radiation Therapy Oncology Group

Time	RT dermatitis grade	HF	CV	p-Value
		Frequency (%)		
Treatment completion	Grade 0	7 (17.5)	4 (10)	0.35
	Grade I	25 (62.5)	23 (57.5)	
	Grade II	8 (20)	13 (32.5)	
One month follow-up	Grade 0	7 (17.5)	2 (5)	0.1
	Grade I	33 (82.5)	36 (90)	
	Grade II	0	2 (5)	
Three months follow-up	Grade 0	22 (56.4)	20 (51.3)	0.82
	Grade I	17 (43.6)	19 (48.7)	
	Grade II	0	0	
Six months follow-up	Grade 0	31 (79.5)	28 (71.8)	0.60
	Grade I	8 (20.5)	11 (28.2)	
	Grade II	0	0	

Radiation pneumonitis was absent at radiotherapy completion in both arms. At one month after treatment conclusion, 5% and 10% of patients had grade I radiation pneumonitis in hypofractionation and conventional arm, respectively (p=0.7), with no incidence of grade II or beyond. At three months, 2.6% vs 12.8% had grade I pneumonitis (p=0.2) in hypofractionation and conventional arm, respectively. At six months 2.6% of patients had grade I radiation pneumonitis in hypofractionation arm, none had it in conventional arm (p=1.0) (Table 3).

**Table 3:** Comparison of radiation pneumonitis. The table shows comparison of radiation pneumonitis at different time points of evaluation between conventional radiotherapy arm and hypofractionated radiotherapy arm. The grading of radiation pneumonitis is as per the RTOG acute toxicity grading. RT pneumonitis: radiation pneumonitis; HF: hypofractionated arm; CV: conventional fractionation arm; RTOG: Radiation Therapy Oncology Group

Time	RT pneumonitis grade	HF	CV	p-Value
		Frequency (%)		
Treatment completion	Grade 0	0	0	-
	Grade I	0	0	
One month follow-up	Grade 0	38 (95)	36 (90)	0.7
	Grade I	2 (5)	4 (10)	
Three months follow-up	Grade 0	38 (97.4)	34 (87.2)	0.2
	Grade I	1 (2.6)	5 (12.8)	
Six months follow-up	Grade 0	38 (97.4)	39 (100)	1.0
	Grade I	1 (2.6)	0	

Dysphagia during treatment was found in 20% of patients in both arms, skin fibrosis at follow-up was found in 30% and 37.5% of patients (p=0.64), lymphedema at follow-up in 7.5% vs 10% of patients (p=1.00), in hypofractionation and conventional arm respectively, shoulder stiffness at follow-up in 2.5% of patients in both arms and no patients had brachial plexopathy at follow-up in both arms (Table 4).

**Table 4:** Comparison of other toxicities.

Toxicity	Hypofractionated arm	Conventional arm	p-Value
	Frequency (%)		
Dysphagia during treatment	8 (20%)	8 (20%)	1.0
Skin fibrosis at follow-up	12 (30%)	15 (37.5%)	0.64
Lymphedema at follow-up	3 (7.5%)	4 (10%)	1.0
Shoulder stiffness at follow-up	1 (2.5%)	1 (2.5%)	1.0
Brachial plexopathy at follow-up	None	None	-

Patient compliance was assessed by analyzing the unplanned gaps. Further, 5% vs 7.5% patients (p=1.0) had unplanned gaps in their radiotherapy schedule in hypofractionation and conventional arm, respectively. The average number of days of interruption were 5.5 vs 8.67 days, respectively (p=0.1).

Quality of life comparison

**Figure 1:**
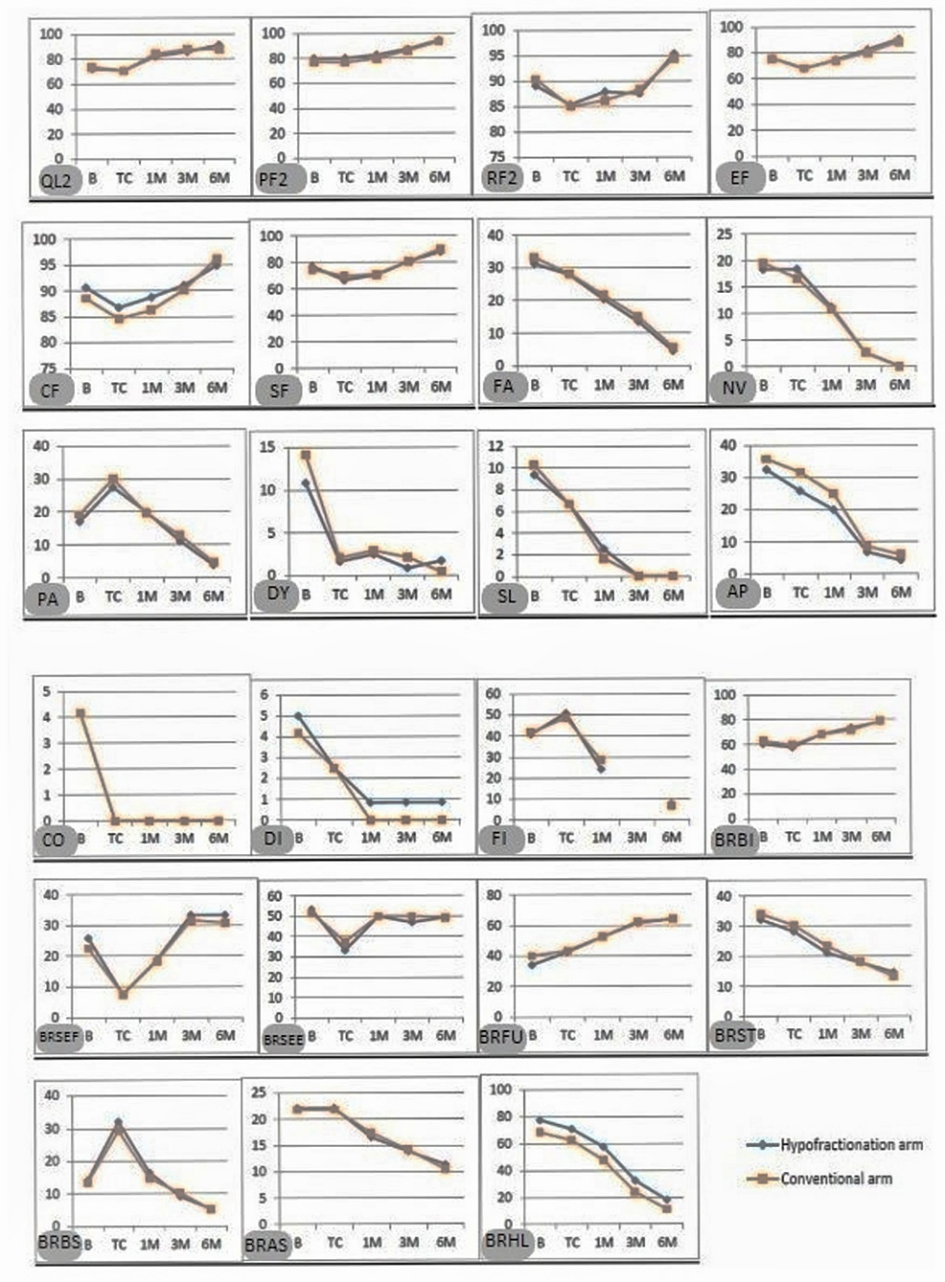
Time trends of quality of life scores at different time points. X-axis: time since baseline to sixth-month follow-up; Y-axis: QOL scores QOL: quality of life; B: baseline; TC: treatment completion; 1M: first-month follow-up; 3M: third-month follow-up; 6M: sixth-month follow-up; QL2: global health status; PF2: physical functioning; RF2: role functioning; EF: emotional functioning; CF: cognitive functioning; SF: social functioning; FA: fatigue; NV: nausea and vomiting; PA: pain; DY: dyspnea; SL: insomnia; AP: appetite loss; CO: constipation; DI: diarrhea; FI: financial difficulties; BRBI: body image; BRSEF: sexual functioning, BRSEE: sexual enjoyment; BRFU: future perspective; BRST: systemic therapy side effects; BRBS: breast symptoms; BRAS: arm symptoms; BRHL: upset by hair loss

All parameters of QLQ-C30 and QLQ-BR23 were comparable at baseline for the two arms (Table 5). The global health status (QL2), functional scales (physical functioning {PF2}, role functioning {RF2}, emotional functioning {EF}, cognitive functioning {CF}, and social functioning {SF}), symptom scales (fatigue {FA}, nausea and vomiting {NV}, pain {PA}, dyspnea {DY}, insomnia {SL}, appetite loss {Ap}, constipation {CO}, diarrhea {DI}, and financial difficulties {FI}), breast cancer-specific functional scales (body image {BRBI}, sexual functioning {BRSEF}, sexual enjoyment {BRSEE}, and future perspective {BRFU}), and breast cancer-specific symptom scales (systemic therapy side effects {BRST}, breast symptoms {BRBS}, arm symptoms {BRAS}, and upset by hair loss {BRHL}) were comparable at baseline and at subsequent evaluations at treatment completion, and at one, three, and six months of follow-up. The trend of the specific scores has been depicted in Figure 1 and is discussed later.

**Table 5:** Comparison of quality of life scores between the two arms at baseline. QOL: quality of life; QLQ-C30: quality of life questionnaire; QLQ-BR23: breast cancer-specific quality of life questionnaire

QOL scale		Hypofractionation arm	Conventional arm	p-Value
QLQ-C30 scales	Global health status (QL2)	72.29	73.88	0.74
	Functional scales			
	Physical functioning (PF2)	79.82	77.49	0.415
	Role functioning (RF2)	89.08	90.36	0.756
	Emotional functioning (EF)	76.03	75.62	0.896
	Cognitive functioning (CF)	90.62	88.54	0.542
	Social functioning (SF)	76.91	74.58	0.584
	Symptom scales			
	Fatigue (FA)	31.26	33.15	0.628
	Nausea and vomiting (NV)	18.32	19.57	0.802
	Pain (PA)	17.09	19.16	0.669
	Dyspnoea (DY)	10.83	14.16	0.609
	Insomnia (SL)	9.40	10.25	0.807
	Appetite loss (AP)	32.49	35.82	0.552
	Constipation (CO)	4.16	4.16	1.00
	Diarrhea (DI)	4.99	4.16	0.749
	Financial difficulties (FI)	40.83	41.66	0.902
QLQ-BR23 scales	Functional scales			
	Body image (BRBI)	60.62	62.91	0.642
	Sexual functioning (BRSEF)	25.83	22.49	0.580
	Sexual enjoyment (BRSEE)	53.32	51.74	0.412
	Future perspective (BRFU)	34.16	39.99	0.446
	Symptom scales			
	Systemic therapy side effects (BRST)	33.23	34.11	0.750
	Breast symptoms (BRBS)	14.16	13.53	0.806
	Arm symptoms (BRAS)	22.07	21.81	0.927
	Upset by hair loss (BRHL)	77.49	68.33	0.815

Comparison of response

At the time of analysis, one patient in conventional arm had local recurrence (chest wall) with distant metastasis as well. Two and five patients, respectively, had distant metastasis in hypofractionation and conventional arm, liver being the most common site of metastasis (four patients), followed by lung (two patients) and bones (one patient).

## Discussion

This prospective randomized study aimed to determine the feasibility and tolerability of hypofractionation over the conventional radiotherapy schedule at a resource-limited rural-based radiotherapy center. Forty patients each in both arms completed treatment. The mean age of the study population was 45.88 years. Corresponding to Kumbhaj et al., an Indian study on hypofractionation, 60.10% of the study population was stage III; however, this could be because we enrolled postmastectomy patients, a high proportion of whom would anyways be locally advanced [13]. In the present study, 52.5% patients had left-sided while 47.5% had right-sided breast cancer, which is in concordance to the available data that says the left-sided breast cancers are 5-10% more than right-sided ones [14]. Sixty-five percent of the study population was hormone receptor-positive, ER positivity being 58.75%, PR positivity 41.25%; 35% human epidermal growth factor receptor-positive. The results correspond to another Indian study by Nandi et al., where 41-43% population (post-breast conservation surgery {BCS} + post-mastectomy) was PR positive and 30-37% were Her2 neu positive, although the ER positivity in their study was higher than ours (91% in BCS cases and 84% in mastectomy cases) [15]. The mean duration of follow-up was 12.48 months.

Incidence of radiation dermatitis appears to favor hypofractionation arm numerically but the difference was found to be statistically insignificant at all time points (Table 2). The rates and grade of skin toxicity were lower in our study compared to other Asian studies. There were higher rates of grade III and beyond acute radiation dermatitis in studies by Kumbhaj et al. (20% vs 5%) for 40 Gy vs 50 Gy, respectively [13], Shahid et al. (37% vs 28% vs 14%) for 27 Gy/week vs 35 Gy/two weeks vs 40 Gy/three weeks [16], and Abhilash et al. (6.6% vs 3.3%) for 39 Gy/13 fractions vs 50 Gy/25 fractions [17]. While in our study, there were no patients with grade III or beyond acute radiation dermatitis, which could be attributed to the fact that bolus was not used for our patients as per institutional protocol and appropriate skincare counseling was timely done and regularly re-enforced. However, there was no significant difference in the incidence of acute radiation dermatitis in any of these studies. Nandi et al. in their study had no incidence of grade III or beyond skin toxicity in concordance to our study [15]. Eldeeb et al. compared three fractionation schedules in post-mastectomy patients, 50 Gy/25 fractions, 45 Gy/17 fractions, and 40 Gy/15 fractions, and found no statistical difference in skin pigmentation between the three arms while grades II-III erythema was significantly higher in the two hypofractionation arms compared to the control arm [18].

The biggest twin trials, the START A and B trials, showed a lower rate of change in skin appearance in hypofractionation patients than the conventional ones. In START A, the event rate when 50 Gy was compared with 41.6 Gy was 31.1% vs 25% and when compared with 39 Gy, it was 31.1% vs 21.6% [4]. In START B trial, the event rate for change in skin appearance was 27.8% vs 22.9% when 50 Gy was compared with 40 Gy [5].

Radiation pneumonitis was overall low (and only limited to grade I) in our study for both arms and was comparable between the two arms at all time points of evaluation (Table 3). Our results are similar to the START trials that provide the most robust evidence in favor of hypofractionation, which showed that radiation pneumonitis with hypofractionation is comparable with the conventional schedule [4,5]. Shahid et al. also noted similar results with statistically insignificant difference in rates of acute radiation pneumonitis between 27 Gy in one week, 35 Gy in two weeks, and 40 Gy in three weeks (4% vs 5% vs 5%).

Dysphagia during treatment was seen in equal proportion (20%) of both groups, with a low overall incidence study compared to other studies where it was nearly 75% patients for Kumbhaj et al. [13], 15-18% for Shahid et al. [16]. For both studies, dysphagia incidence was comparable between the two arms. Skin fibrosis until analysis (30% vs 37.5%) was comparable between the hypofractionated and conventional arms. Lymphedema (7.5% vs 10%) was comparable between hypofractionated and conventional arms, with similar results by Kumbhaj et al. (7% vs 2%); however, Shahid et al. found higher rates of lymphedema (35-41%) [13,16]. Shoulder stiffness was in equal proportion (2.5%) of both the groups, as for Kumbhaj et al. (2% had shoulder restriction in each arm), although Abhilash et al. had higher rates (20% vs 30%) in hypofractionation and conventional arms but with statistically insignificant differences [13,17]. None had brachial plexopathy in both the arms until analysis as in START B trial, where no brachial plexopathy was seen in 82 and 79 patients (axilla and SCF treated) in hypofractionation and conventional arm, respectively [5].

Five percent vs 7.5% of patients had unplanned gaps in hypofractionated and conventional arm, respectively, with insignificant difference, the average number of days of interruption being higher in hypofractionated arm (5.5 vs 8.66 days) although statistically insignificant. This higher number of days of unplanned interruption for conventional arm could be attributed to the higher cost of not only the treatment per se but also of traveling and lodging, but to establish this fact, larger sample size needs to be studied. In this study, 97.5% of patients (78 out of 80) completed the EORTC QOL questionnaires at all points of evaluation. In the START B trial, the compliance with completion of quality of life questionnaires over five years was more than 90%.

Global health status (QL2), as well as all parameters of QLQ-C30 and QLQ-BR23, was comparable at baseline for the two arms (Table 5). QL2 of patients in this study were comparable at all time points of evaluation and showed a decreasing trend attributable to the trend of functional and symptom scales, as seen in Figure 1, and is discussed below.

When trend over time, since baseline was evaluated, we observed that the physical functioning (PF2) score was almost the same at baseline and at the completion of treatment, after which during subsequent follow-ups, it showed an upward trend for both arms (Figure 1). The low PF2 score at baseline could be because all patients had completed their last course of chemotherapy almost three weeks before starting radiotherapy, and they were likely to have generalized weakness associated with chemotherapy at that time. Once started on radiotherapy, the physical functioning was restricted due to hospital stay, or daily traveling for radiotherapy which itself leads to fatigue. After radiotherapy completion, when patients came for follow-up after one month, for most patients, there was improvement in their ability to be able to take a walk, perform self-care, or do strenuous work.

Role functioning score (RF2) showed a dip from baseline to the end of radiotherapy for patients of both arms, as during radiotherapy, activities like pursuing their hobbies and performing other work are likely to be restricted due to a large proportion of time spent at either the radiotherapy department or during traveling. Even for patients staying in the hospital being able to perform the above activities in a foreign environment is difficult. Once radiotherapy was completed, for the same reason, the score shows a rise at subsequent follow-ups when the general well-being of the patients improved and side effects due to chemotherapy and radiotherapy faded out (Figure 1).

Emotional functioning score (EF) was again comparable at all time points of evaluation for both arms (Figure 1). For both arms, patients showed a dip in the score after radiotherapy starting until completion which could be attributed to feelings of tension, worriedness, depression during radiotherapy which consumes a patient not only physically but drains one emotionally as well. To travel every day and reach the radiotherapy center on their scheduled time for three to five weeks adds to the stress of losing work hours for themselves and for the accompanying person, which in most cases is the husband or son, who is the earning head. The emotional score after completion of radiotherapy showed a gradual increase during subsequent follow-ups, when the stress is bound to decrease as patients would be staying at home with a slow return to their normal routine.

Cognitive functioning score was comparable at baseline for both the arms and at subsequent evaluation at all time points. Surprisingly, it also showed an increasing trend over time since the completion of radiotherapy (Figure 1). Social functioning score (SF) was again comparable at baseline, and then at all points of evaluation. The score showed a decrease from baseline till radiotherapy completion when for obvious reasons, the daily radiotherapy schedule interfered with the patients’ family life and social life. After completion of radiotherapy, when patients went back home, the score showed an upward trend (Figure 1).

Symptom scales

Fatigue (FA), nausea and vomiting (NV), pain (PA), dyspnea (DY), insomnia (SL), appetite loss (AP), constipation (CO), and diarrhea (DI) were all comparable at baseline and all time points of evaluation (Table 5, Figure 1). Fatigue, nausea and vomiting, insomnia, appetite loss showed a gradual decrease from baseline until the last time point of evaluation (six months follow-up). This could be attributed to the recovery of patients from chemotherapy-related side effects and patients might not have been affected by radiotherapy with respect to these parameters. While studying the trend of pain, we observed that it increased from baseline to the end of radiotherapy, after which it showed a downward trend. Dyspnea score showed a big dip from baseline to treatment completion, after which it was more or less the same over time. Constipation (CO) and diarrhea (DI) scores were low overall and decreased from baseline to further time points, showing that these symptoms were not affected by radiotherapy. The slightly higher score for these symptoms present at baseline could be a result of the prior cycles of chemotherapy, the effect of which faded over time. The parameter of financial difficulties (FI) was comparable at baseline and at all time points of evaluation. The score showed an upward trend from baseline till radiotherapy completion, which is attributable to the money spent in treatment per se as well as for stay and travel. After completion of radiotherapy, the score showed a gradual downward trend with time.

Functional scales (when dealing with breast cancer-specific quality of life)

Body image (BRBI), sexual functioning (BRSEF), sexual enjoyment (BRSEE), and future perspective (BRFU) were comparable at baseline as well as all further time points of evaluation (Table 5, Figure 1). The body image scale was low and almost similar at baseline and at treatment completion. After the first follow-up, it showed a gradual upward trend for all patients. This could be due to a better acceptance of their body image after resolution of the skin reactions during and after the first follow-up when patients must have started feeling attractive and feminine again. Sexual functioning scale (BRSEF) and the associated sexual enjoyment scale (BRSEE) for both arms were observed to show a dip from baseline till treatment completion, after which it was seen to rise with time over subsequent follow-ups.

Breast cancer-specific symptom scales

Systemic therapy side effects (BRST), breast symptoms (BRBS), arm symptoms (BRAS), and upset by hair loss (BRHL) are comparable at baseline as well as all further time points of evaluation (Table 5, Figure 1). Systemic therapy side effects decreased over time from baseline for obvious reasons of recovery from side effects of chemotherapy over time. The breast symptom evaluation score increased from baseline to treatment completion, which is attributable to radiotherapy side effects of pain, swelling, increased sensitivity, and dryness/itchiness over the radiotherapy site. Once radiotherapy was completed, the score should a downward trend with time over subsequent follow-ups as the above-mentioned side effects recovered. The arm symptom score was similar at baseline and at treatment completion, after which it showed a downward trend over time at subsequent follow-ups, which is attributable to gradual recovery from radiotherapy-induced pain and swelling over axilla. The score for upset from hair loss also decreased from baseline with time which can be explained by gradual regrowth of hair lost due to chemotherapy.

The quality of life subgroup in START B trial included 12.3% post-mastectomy women for whom the relevant parameter in QOL assessment changed in skin appearance which favored the hypofractionation arm with a hazard ratio of 0.86 on the forest plot [5]. START A trialists observed a lower rate of change in skin appearance after 39 Gy than 50 Gy (p=0.004) [4]. We observed comparable scores for breast symptoms of pain, itching, and swelling in the irradiated between two arms at all time points of evaluation.

Efficacy of hypofractionation was not undertaken as an objective for this study, owing to a too short follow-up to be able to draw conclusions on it. The higher number of distant metastasis in the conventional arm (5 vs 2) will have to be considered as an incidental finding because follow-up duration is very short to make any inference on the same. Small sample size and a short follow-up duration limit our study results. Longer follow-up on a large sample (multicentric trial) is required to analyze the response and the late cardiac effects.

## Conclusions

Hypofractionation with 40 Gray/25 fractions/five weeks in post-mastectomy setting is feasible and non-inferior to conventional five-week fractionation schedule in terms of toxicity profile and quality of life for our patients. Efficacy of the same has already been established by the START trials in the United Kingdom. A three-week schedule costs 0.6 times less compared to the five-week schedule. A treatment modality that costs less and offers similar toxicity profile, quality of life, and efficacy should be readily accepted. A shorter schedule would also lessen the burden on the radiotherapy machines which would in turn shorten the waitlists. Hence, resource constraint institutes in India can adopt hypofractionation as their post-mastectomy radiotherapy protocol, which not only improves patient compliance but also saves resources for both patients and the institute.
